# High prevalence of the *MLH1 V384D* germline mutation in patients with HER2-positive luminal B breast cancer

**DOI:** 10.1038/s41598-019-47439-3

**Published:** 2019-07-29

**Authors:** Seung Eun Lee, Hye Seung Lee, Kyoung-Yeon Kim, Jung-Hoon Park, Hanseong Roh, Ha Young Park, Wan-Seop Kim

**Affiliations:** 1 Department of Pathology, Konkuk University Medical Center, Konkuk University School of Medicine, Seoul, Korea; 2 Precision Medicine Institute, Macrogen, Inc., Seoul, Korea; 3 Department of Pathology, Busan Paik Hospital, Inje University College of Medicine, Busan, Korea

**Keywords:** Breast cancer, Cancer genomics, Medical genomics

## Abstract

HER2-positive luminal B breast cancer (BC), a subset of the luminal B subtype, is ER-positive and HER2-positive BC which is approximately 10% of all BC. However, HER2-positive luminal B BC has received less attention and is less represented in previous molecular analyses than other subtypes. Hence, it is important to elucidate the molecular biology of HER2-positive luminal B BC to stratify patients in a way that allows them to receive their respective optimal treatment. We performed molecular profiling using targeted next-generation sequencing on 94 HER2-positive luminal B BC to identify its molecular characteristics. A total of 134 somatic nonsynonymous mutations, including 131 nonsynonymous single nucleotide variants and three coding insertions/deletions were identified in 30 genes of 75 samples. *PIK3CA* was most frequently mutated (38/94, 40.4%), followed by *TP53* (31/94, 33.0%), and others were detected at lower frequencies. Recurrent germline mutations of *MLH1* V384D were found in 13.8% (13/94), with a significantly high *TP53* mutations rate. The frequency of *MLH1* V384D germline mutation in individuals with HER2-positive luminal B BC was significantly higher than that observed in the controls. All 13 cases were classified as microsatellite stable tumors. Tumor mutation burdens (TMB) were not significantly different between *MLH1* V384D carrier and wild type. The concordant results of microsatellite instability (MSI) and TMB suggest that the haploinsufficiency of *MLH1* plays a role as a tumor predisposition factor rather than a direct oncogenic driver. Our study identified, for the first time, that *MLH1* V384D germline variant is frequently detected in HER2-positive luminal B BC. *MLH1* V384D germline variant may not only contribute to gastrointestinal cancer predisposition but may also contribute to BC in East Asians.

## Introduction

Breast cancers (BC) are incredibly heterogeneous diseases in terms of the mutations, structural variations, and copy number aberrations they harbor. Genomic and molecular profiling analyses have extensively advanced our understanding of BC biology and have increased the elucidation of its five intrinsic molecular subtypes (luminal A, luminal B, HER2-enriched, basal-like, and normal breast like) by genome-wide expression analyses^1,2^. In intrinsic molecular subtypes, luminal B subtype is clinically and molecularly heterogeneous group. HER2-positive luminal B group, a subset of the luminal B subtype is ER-positive and HER2-positive BC, which is approximately 10% of all BC^3,4^. However, HER2-positive luminal B BC has received less attention and been less represented in previous molecular analyses than other subtypes. HER2-positive luminal B positive BC has been shown to be a heterogenous group^5^. Hormone receptor and HER2 co-expression may partially account for heterogeneity in the HER2-positive luminal B positive group. HER2 and ER pathways are strictly related in a bi-directional way^6^. HER2 signaling causes endocrine resistance, and ER modulates the response, not only to chemotherapy, but also to HER2 targeted therapy^6^, which is a major challenge in the treatment of HER2-positive luminal B BC. Therefore, it is important to elucidate the molecular biology of HER2-positive luminal B BC to stratify patients so that each can receive the optimal treatment.

More recently, using multi-omics profiling, analysis of a Korean BC cohort enriched with younger, premenopausal patients was found to harbor significant molecular differences from the TCGA cohort enriched with Western post-menopausal patients^7^. Korean BC cohort had significantly higher proportion of the HER2-positive luminal B BC subtype than TCGA cohort (16.1 vs 5.4%). Furthermore, there are also differences in molecular features and tumor immune microenvironments between Asian and Western BC.

Here we report a study in which molecular profiling is performed using targeted next-generation sequencing (NGS) of 94 HER2-positive luminal B BCs to identify the molecular characteristics of HER2-positive luminal B BC in East Asians.

## Results

### Clinicopathologic characteristics of 94 HER2-positive luminal B breast cancer patients

A total of 94 HER2-positive luminal B BC samples (KF) were analyzed in this study. The clinicopathologic characteristics are presented in Table 1 and Supplementary Table 1. The median age at diagnosis was 49 years (range, 25–75). Of patients, 52 (55.3%) were premenopausal, and 41 (43.6%) were post-menopausal. The most common histologic type was invasive ductal carcinoma (95.7%). Of these 94 patients, 72 (76.6%) received adjuvant trastuzumab, 80 (85.1%) received adjuvant chemotherapy, 72 (76.6%) received hormonal therapy, and 77 (81.9%) received radiation therapy. Eight of the 94 patients (8.5%) showed recurrence. The median time to recur after curative resection was 16.5 months and range was from 2 to 53 months.

**Table 1:** Clinicopathologic characteristics of 94 HER2-positive luminal B breast cancer patients’.

Characteristics	No. of patients (%)
**Age (years)**	
Median (range)	49 (25–75)
**Menopausal status**	
Pre-menopause	52 (55.3)
Post-menopause	41 (43.6)
N/A	1 (1.1)
**Histologic type**	
IDC	90 (95.7)
ILC	1 (1.1)
Mucinous carcinoma	2 (2.1)
Micropapillary carcinoma	1 (1.1)
**T-stage**	
1	43 (45.7)
2	48 (51.1)
3	3 (3.2)
**N stage**	
0	50 (53.8)
1	28 (30.1)
2	12 (12.9)
3	3 (3.2)
N/A	1 (1.1)
**Stage**	
I	32 (34.0)
II	45 (47.9)
III	16 (17.1)
IV	1 (1.1)
**Histologic grade**	
I	2 (2.1)
II	33 (35.1)
III	59 (62.8)
**Herceptin Tx**	
Yes	72 (76.6)
No	17 (18.1)
NA	5 (5.3)
**Chemo Tx**	
Yes	80 (85.1)
No	9 (9.6)
NA	5 (5.3)
**Hormonal Tx**	
Yes	72 (76.6)
No	17 (18.1)
NA	5 (5.3)
**Radiation Tx**	
Yes	77 (81.9)
No	12 (12.8)
NA	5 (5.3)
**Recur**	
Yes	8 (8.5)
No	81 (86.2)
NA	5 (5.3)

### Genomic profiling in HER2-positive luminal B breast cancer

**Figure 1:**
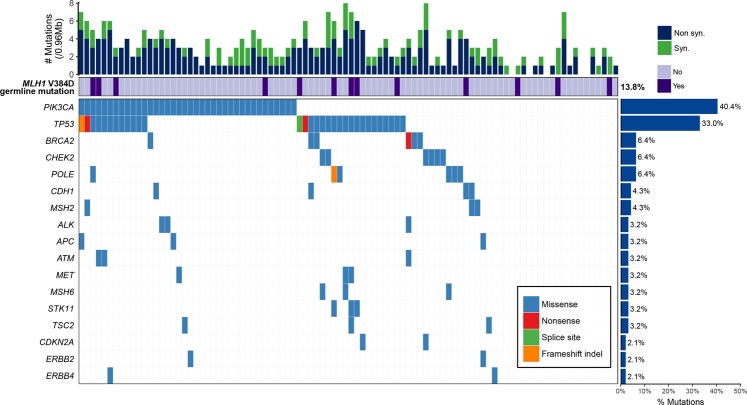
Recurrently mutated genes in HER2-positive luminal B BC. Each column represents a case. The top panel shows tumor mutation burden. The middle panel shows *MLH1* V384D germline mutation status: dark purple, mutant type; light purple, wild type. The bottom panel shows distribution of mutations. Four mutation types are distinguished by different colors. The right panel represents the mutation frequency in the 94 samples.

We sequenced 170 cancer-related genes with FFPE tumor tissue of 94 HER2-positive luminal B BC. Targeted sequencing summary is shown in Supplementary Table 4. A total of 134 somatic nonsynonymous mutations, including 131 nonsynonymous single nucleotide variants (nsSNVs) and three coding insertions/deletions (indels), were identified in 30 genes of 75 samples. A full list of variants is shown in Supplementary Table 5. Figure 1 depicts sequence variants of 17 genes detected in more than two samples. The most frequently mutated was *PIK3CA* (38/94, 40.4%), followed by *TP53* (31/94, 33.0%), with the others detected at lower frequencies. All *PIK3CA* variants were missense, and they occurred mostly in two hotspots (34/38, 89.5%) located in the helical and kinase domains (exon 9 and exon 20), resulting in activation of the gene with constant auto-phosphorylation. The majority of *TP53* mutations were missense (27/31, 87.1%), and the remaining were truncating. The nature of tumor suppressor was reflected by the fact that the loci of the variants were scattered. When compared with The Cancer Genome Atlas (TCGA) and Molecular Taxonomy of Breast Cancer International Consortium (METABRIC), the frequency of *PIK3CA* mutations was higher in KF (40.4%) than TCGA (25.0%) and METABRIC (23.8%). If variants with low allele frequency (AF) (<5%) were included, the difference achieved statistical significance (*p* = 0.01). On the other hand, the frequency of *TP53* was significantly lower (*p* = 0.01) in KF (33.0%) than TCGA (42.3%) and METABRIC (54%) (Supplementary Fig. 1). We checked total read depth (TR) and variant allele frequency (VAF) of the TCGA and METABRIC BC data set. The median VAF was 33.7, 36.2, and 16.1 for TCGA, METABRIC, and KF, respectively, while the median TR was 72x, 176x, and 752x (Supplementary Fig. 2). VAF and TR were significantly different among three groups (*p* < 2.2e-16). Since we sequenced relatively high depth with median mean depth 1578x, we found more subclonal events that might have been missed in previous studies. Among the variants with recurrent positions shared by more than two samples, others than *PIK3CA* and *TP53*, were mostly “uncertain significance” of ClinVar annotation. *CDH1* p.V832M, *CHEK2* p.H371Y, and *ERBB2* p.V747L were “likely pathogenic” or “pathogenic” in ClinVar annotation. The others were annotated as “uncertain significance” in ClinVar. Unfortunately, genes frequently mutated in HER2-positive luminal B BC in TCGA and METABRIC (*GATA3*, *ARID1A*, and *KMT2C*) were not included in our gene panels. Their mutation frequency could not be assessed.

### Frequent *MLH1* mutations in HER2-positive luminal B breast cancer

**Figure 2:**
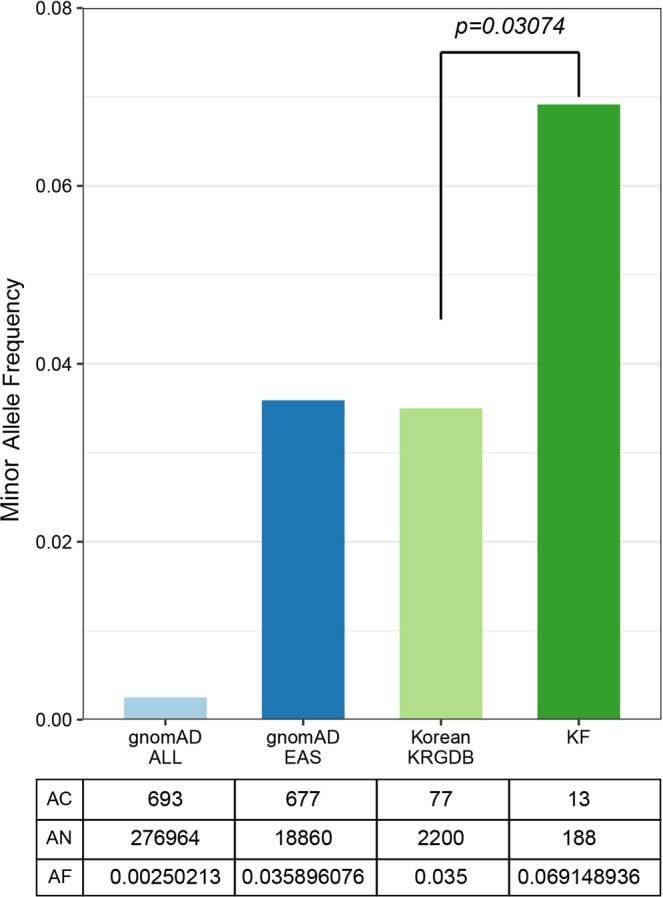
Different minor allele frequencies among four populations. Bar plot compares minor allele frequency of HER2-positive luminal B BC with three non-cancer populations, gnomAD all, East Asian (EAS), non-cancer Korean (KRGDB) and present study (KF). The bottom table represents each allele number (AN), allele count (AC), and allele frequency (AF).

Although there is a slight difference in mutation frequencies of *PIK3CA* and *TP53*, we obtained similar mutation profiles as the previous large-scale studies. To identify other novel findings within our data, we focused on rare germline mutations. The probable germline mutations could not be perfectly filtered out in clinical cancer genome analysis, since matched normal samples usually are not examined. Under the same condition, rare germline mutations with clinical significance could be included in the present study. Therefore, we reviewed variants with minor allele frequency (MAF) between 1–5% in gnomAD EAS, which was excluded to filter out putative germline polymorphism. Interestingly, recurrent *MLH1* mutations on chr3:37067240 (p.V384D) were identified in 13 samples (prevalence: 13.8%, MAF: 0.069). Thirteen HER2-positive luminal B BC tissues revealed a *MLH1* V384D with median 45.09% VAF (range: 31.8–60.12). The median of depth of coverage in the region is 1273 (range: 69–2121). The MAFs of that position were 0.003 and 0.036 in gnomAD All and EAS, respectively. We checked MAF of *MLH1* V384D in non-cancer Korean database of 1100 individuals (http://152.99.75.168/KRGDB/menuPages/intro.jsp). The frequency was 0.035, which is almost identical to gnomAD EAS and about one half the value of HER2-positive luminal B BC. The frequency of the *MLH1* V384D mutation in individuals with HER2-positive luminal B BC was significantly higher than that observed in the controls (Fig. 2). We compared mutational frequencies of *MLH1* V384D with those of HER2-negative luminal type BC and triple negative type BC (in-house data, not published). The frequency was 8.2% (19/233) in HER2-negative luminal type and 4.8% (5/104) in triple negative type. We have no available data of HER2-positive luminal B BC, the frequency of *MLH1* V384D for that group could not be assessed. *MLH1* V384D mutation might be associated with ER positivity, further study is needed to check its relationship with HER2 positivity.

### Validation for *MLH1* V384D mutation

**Figure 3:**
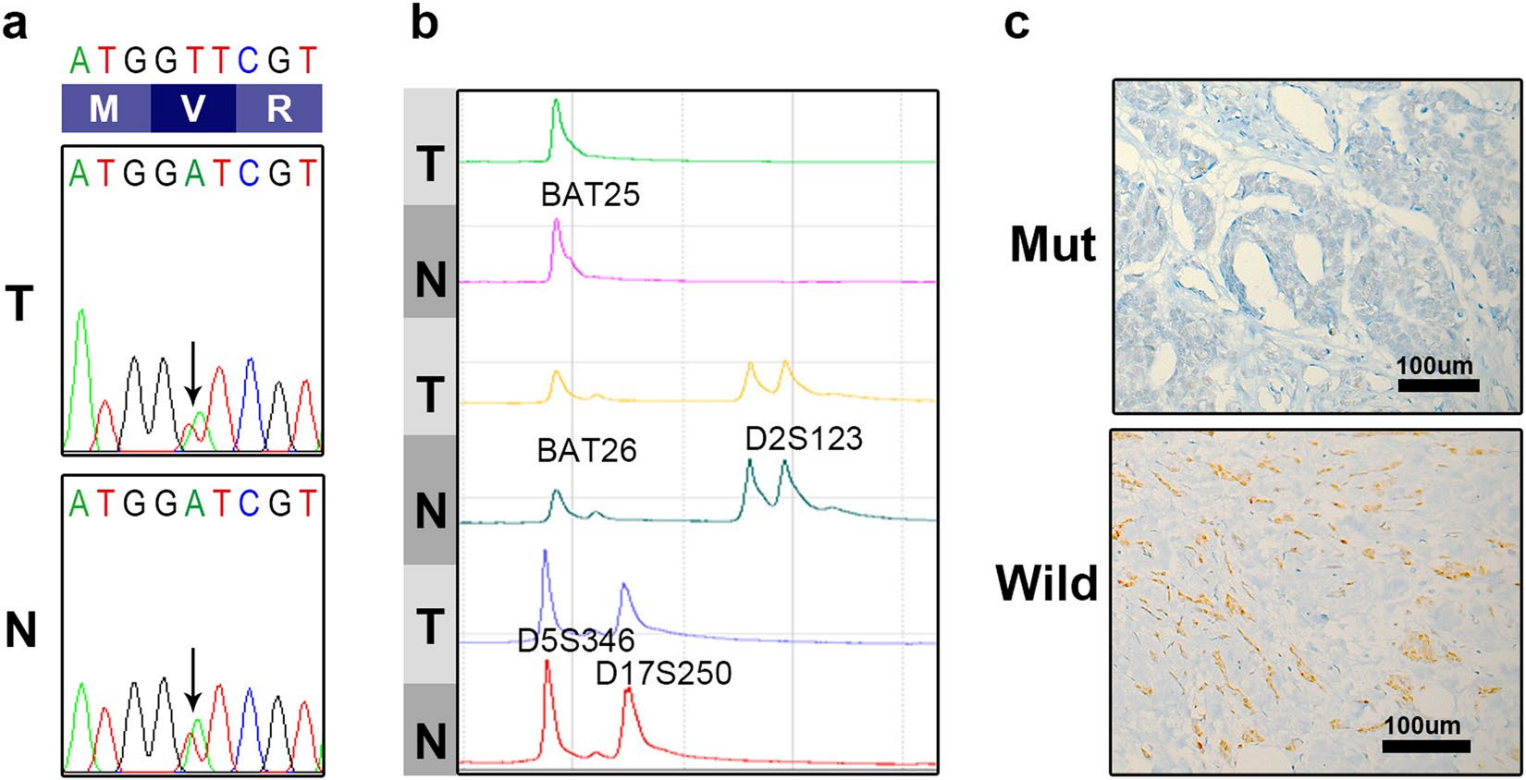
Representative results of Sanger sequencing, MSI and immunohistochemical analysis of MLH1 expression of a germline *MLH1* V384D mutant case. (**a**) Sanger sequencing of *MLH1* V384D for tumor and matched normal revealed that it is germline heterozygous variant. (**b**) MSI profiles with five Bethesda markers in the same case demonstrated MSS. (**c**) Representative cases with focal loss of MLH1 expression in a mutant case and intact MLH1 expression in a wild-type case. Abbreviations: T = tumor tissue; N = normal tissue.

To check whether *MLH1* V384D was a germline or somatic, we performed conventional Sanger sequencing in each tumor and matched normal breast tissues. The variant of c.1151T > A (V384D) was detected in all 13 pairs of tumor and normal samples (Fig. 3a). We confirmed these *MLH1* V384D mutations are germline heterozygous mutation.

Microsatellite instability (MSI) analysis was performed in all 13 cases. None of the cases revealed instability at any of the five loci analyzed, and they were classified as microsatellite stable tumor (MSS, 0/5) (Fig. 3b). To evaluate the functional loss of MLH1 protein, we performed immunohistochemical analysis for MLH1 protein. In three of 13 cases with *MHL1* mutation, both carcinoma and adjacent normal breast tissue showed an area of focal loss, while all 81 cases without MLH1 mutation showed uniform positive staining (Fig. 3c).

### *MLH1* V384D in patients with 13 HER2-positive luminal B breast cancers

The 13 patients included 12 females and one male. Of them, six patients (46.2%) were premenopausal and six (46.2%) were postmenopausal woman. The detailed clinical and pathologic findings are summarized in Supplementary Table 6. Histologic types were invasive ductal carcinoma of no special type in 12 cases and mucinous carcinoma in one case. Tumor stage was IA in four cases, stage IIA in five cases, and stage IIB in four cases by AJCC eighth edition. None of the patients had any recurrence. Table 2 shows a comparison between the clinicopathologic and molecular data of the 13 patients with the *MLH1* V384D germline mutation and those of the 81 patients with wild *MLH1*. The two groups were statistically similar with regard to age, menopausal status, T stage, N stage, AJCC stage, histologic grade, *PIK3CA* mutation status, and recurrence. The frequency of *TP53* mutation in the *MLH1* V384D germline mutation group was significantly higher than that of the wild *MLH1* group (*p* = 0.03). Tumor mutation burdens (TMB) were not significantly different between the two groups, which is concordant with the result of MSI analysis.

**Table 2:** The clinicopathologic data of 13 patients with *MLH1* V384D germline mutation compared with 81 patients with wild *MLH1*.

	*MLH1* wild	*MLH1* V384D	p-value
	(N = 81, 86.2%)	(N = 13, 13.8%)	
Age	48 (25–74)	52 (42–71)	0.145
Menopausal status			0.134
Pre	46 (56.8%)	6 (46.2%)	
Post	35 (43.2%)	6 (46.2%)	
N/A	0	1 (7.7%)	
T_stage			0.517
1	37 (45.7%)	6 (46.2%)	
2	42 (51.9%)	6 (46.2%)	
3	2 (2.5%)	1 (7.7%)	
N_stage			0.496
0	43 (53.1%)	8 (61.5%)	
1	23 (28.4%)	5 (38.5%)	
2	12 (14.8%)	0	
3	3 (3.7%)	0	
Stage			0.262
1	28 (34.6%)	4 (30.8%)	
2	36 (44.4%)	9 (69.2%)	
3	16 (19.8%)	0	
4	1 (1.2%)	0	
Histologic grade			0.188
1	1 (1.2%)	1 (7.7%)	
2	30 (37.0%)	3 (23.1%)	
3	50 (61.7%)	9 (69.2%)	
*PIK3CA* mutation			0.774
Not identified	46 (56.8%)	8 (61.5%)	
Present	35 (43.2%)	5 (38.5%)	
*TP53* mutation			0.027
Not identified	58 (71.6%)	5 (38.5%)	
Present	23 (28.4%)	8 (61.5%)	
Recurrence			0.651
No	68 (84.0%)	13 (100%)	
Yes	8 (9.9%)	0	
N/A	5 (6.2%)	0	

## Discussion

The present study presents molecular profiling in 94 HER2-positive luminal B BCs using a targeted next-generation sequencing assay. We sequenced 170 cancer-related genes, and variants were shown to be enriched in *PIK3CA* and *TP53*. Recurrent germline mutations of *MLH1* were found in 13.8%, with a significantly high *TP53* mutations rate.

The two top-ranked genes were *PIK3CA* and *TP53*, and variants in the other genes exhibited long tail distribution. These results are consistent with those of previous studies on molecular analysis of patients with BC^8,9^. The prevalence of *PIK3CA* mutations has been variously reported in accordance with the different biologic subtypes of BC, which tends to be more common in hormone receptor positive BC (35%) than HER2 positive BC (22–31%) and triple negative BC (8.3%)^8,9^. When compared with TCGA and METABRIC HER2-positive luminal B BC, the *PIK3CA* mutation frequency was higher in KF population. But considering VAF, our results included subclonal events with low VAF, which might be missed in the TCGA and METABRIC. This is supported by a recent research that also revealed a comparably high mutation rate of *PIK3CA* (22.1%, 23/104) in ultradepth sequencing of triple-negative BC^10^. *PIK3CA* mutant group are associated with low chemosensitivity and resistance to anti-HER2 therapy in metastatic HER2-positive luminal B BC^11,12^. *TP53* mutations are associated with triple-negative BC group, more aggressive subtypes and resistance to chemotherapies^13^. In a recent study, the significance of *TP53* as a prognostic marker in BC was different in a molecular subgroup^14^. They reported that *TP53* mutations were independent poor prognostic marker in luminal B, HER2-enriched groups^14^. In our study, *MLH1* mutant group had significantly high *TP53* mutation rate. Although no recurrence identified in *MLH1* mutant group, it is necessary to confirm the relationship of *MLH1* mutation and prognosis in a larger sample group.

Interestingly, we found that 13.8% of patients carried the *MLH1* V384D germline mutation of the 94 HER2-positive luminal B BC. *MLH1* is a tumor suppressor gene involved in DNA mismatch repair (MMR)^15^. *MLH1* germline mutations are known to cause Lynch syndrome^16^. The most common malignant tumors in Lynch syndrome are colorectal and endometrial carcinoma^17^. In addition to germline mutations, somatic mutations in *MLH1* have been identified in colorectal and endometrial carcinomas^18^. Although the *MLH1* mutation was generally known to occur in the gastrointestinal cancer susceptibility syndrome, several studies suggested that *MLH1* mutation carriers have increased risk of various cancers, such as renal, pancreatic, urinary bladder, prostate, and female BC^12,19–21^. In particular, there was controversy as to whether the MMR deficiency increased BC^22–26^. One study prospectively stated that the risk of BC was increased about four-fold above that of the control group^19^. More recently, another study found 10 *MLH1* germline mutations in 8085 unselected Chinese BC patients, including 965 HER2-positive luminal B BC patients^27^. However, there was a report of 25 BC susceptibility genes in 488 patients of a Western BC population, including 63 HER2-positive luminal B BC patients, which were not found to have a germline *MLH1* mutation^28^.

MLH1 dimerizes with PMS2 to form MutL heterodimer, which plays an important role in the MMR system^29^. The V384D mutation is likely to impair the stability of the hMLH1 protein, which can eventually partially reduce the interaction between hMLH1 and hPMS2^30^. Finally, it was demonstrated that the V384D mutation reduced MMR activity *in vitro*^31^. Therefore, the *MLH1* V384D mutation is partly a loss of function variant. In our study, we found that the *MLH1* V384D mutation is a heterozygous germline mutation.

A heterozygous germline mutation of tumor suppressor gene allele establishes a predisposition to develop cancer^32^. The mechanism to explain the heterozygous effect is haploinsufficiency^32^. These effects can be directly attributable to the reduction in gene dosage or can work in cooperation with other tumorigenesis or haploinsufficient events^32^. Heterozygous *MSH2* mutant mice appear to be more susceptible to tumor formation than wild-type animals^33–35^. *MSH2* haploinsufficiency might have phenotypic effects that could contribute to progression of cancers in HNPCC individuals^33–35^. That is why haploinsufficiency might increase the cancer risk in *MLH1* V384D mutation carrier^30^.

In our study, the frequency of the *MLH1* V384D germline mutation in the HER2-positive luminal B BC group was two times higher than the mutation rate in both East Asian and Korean patients. Few published studies in East Asia have been about *MLH1* V384D germline mutation in sporadic cancer. Ohasawa *et al*.^36^ stated that *MLH1* V384D mutant was detected in 6% of 670 colorectal cancer patients, but it was only detected in 1.5% of 322 patients in the control group in the Japanese population. Peng *et al*.^37^ reported that a *MLH1* V384D mutant was significantly higher in a colorectal cancer group than in the 311 members of a control group (9.0% vs 0.3%) in the Chinese population. Recently, Chiu *et al*.^38^ reported that the *MLH1* V384D allele was overrepresented in *EGFR* L858R-positive lung adenocarcinoma patients with epidermal growth tyrosine kinase inhibitor (EGFR-TKI) resistance. Therefore, ethnic differences are evident the frequency of *MLH1* V384D mutation between East Asian and Western populations.

We also identified that the frequency of the *TP53* mutation was significantly high in the *MLH1* V384D germline mutation carrier. Several studies have demonstrated that malignancy arising in carriers of *BRCA1*/*BRCA2* germline mutations showed the higher rate of *TP53* mutations^39,40^. These observations suggest that the loss of p53 function may be a critical genetic event of tumorigenesis in background of a germline *BRCA1* / *BRCA2* mutation. Consistent with this theory, our results can be explained in this context.

Few studies have investigated the germline mutations in BC susceptibility genes^27,28^. Germline mutations particularly for *BRCA1* mutations are significantly associated with triple-negative BC phenotype among the four molecular subgroups^27^, which indicated that triple-negative BC reveals high level of genomic instability and a more aggressive phenotype. On the other hand, HER2–positive BC exhibits fewer germline mutations than other molecular subgroups^27^. This is explained by the fact that *HER2* is a potent driver gene that could initially induce tumorigenesis^41^. In our study, we did not perform these analyses in other BC molecular subgroups. It is yet unknown whether *MLH1* V384D germline mutation was significantly different among other molecular subgroups. Therefore, additional studies for more diverse unselected BC are needed to establish this.

It is now well-known that MMR-deficient tumors exhibit a high tumor mutation burden (TMB). Several studies assessed microsatellite instability using TMB^42,43^. In our study, there was no significant different between *MLH1* V384D mutant and the wild group with regard to TMB, and all members of the *MLH1* V384D mutant group showed MSS in conventional MSI analysis. Cancers with MSI-H phenotype in Lynch syndrome develop when second hit mutation occurs in wild-type allele followed in addition to pre-existing germline mutation^44^. The concordant results of MSI analysis and TMB suggest that the haploinsufficiency of *MLH1* plays a role as a tumor predisposition factor rather than a direct oncogenic driver.

In conclusion, our study identified, for the first time, *MLH1* V384D germline variant, which is frequently detected in HER2-positive luminal B BC. *MLH1* V384D germline variant may not only contribute to gastrointestinal cancer predisposition but may also contribute to BC in East Asians. Further investigation into the role of *MLH1* V384D germline mutation in BC will be needed to identify the association between MMR genes and BC risk.

## Materials and Methods

### Patients

This cohort study with retrospective data evaluated clinical, histological, and immunohistochemical charcteristics of all patients with histologically confirmed 94 HER2-positive luminal B BC (Supplementary Table 1). The cohort was composed of Korean non-related individuals. The samples were obtained from patients who underwent surgical resection for primary BC at the Konkuk University Medical Center from January 2012 to December 2016. All patients provided informed consent before surgical resection. Study approval was obtained from the Institutional Review Board of Konkuk University Medical Center (KUH1210049) and all experiments were performed in accordance with the approved guidelines and regulations.

Medical records and H&E-stained sections were reviewed to obtain clinicopathologic information. The following histopathologic variables of the invasive carcinomas were determined: histologic subtype, T stage, N stage, AJCC stage, Bloom-Richardson histologic grade. Samples with tumor cellularity >50% were included in this study.

### Targeted Next-generation sequencing

Using the Custom Cancer Panel (Agilent Technologies, Inc., Santa Clara, California, USA) after DNA isolation from formalin-fixed, paraffin-embedded (FFPE) samples, we sequenced 170 cancer-related genes (Supplementary Table 2) to identify genetic mutations in 94 samples from HER2-positive luminal B BC patients. A dsDNA HS Assay Kit (Life Technologies, Grand Island, NY) was used to quantify DNA samples by Qubit 2.0. Sequencing libraries were prepared with SureSelectXT Library Prep Kit (Agilent Technologies, Inc., Santa Clara, California, USA) as previously decribed^45^. In brief, 200 ng of genomic DNA from the FFPE samples was fragmented by Covaris E220 instrument (Covaris, Woburn, MA) and then subjected to end repair, A-Tailing, and adapter ligation. Agencourt AMPure XP beads (Beckman Coulter, Beverly, MA) were used to remove unligated adaptors. The resulting libraries were PCR-amplified and purified with Agencourt AMPure XP beads. Captured libraries were PCR-amplified using Illumina p5 and p7 primers and purified with Agencourt AMPure XP beads. Library was quantified using a KAPA Library Quantification Kit (KAPA Biosystems), and its fragment size was analyzed by Bioanalyzer 2100 (Agilent Technologies, Cedar Creek, TX). Once ready, libraries were sequenced on Illumina HiSeq2500 platforms (Illumina, San Diego, CA).

### Identification of somatic mutations

FASTQ file quality control was performed using FastQC v0.11.5 (http://www.bioinformatics.babraham.ac.uk/projects/fastqc/) software. The adapter sequences were removed by cutadapt v1.9.1.^46^. Sequencing reads were mapped to Human Genome version 19 (hg19) using the Burrows-Wheeler Aligner^47^. Poorly mapped reads that had mapping quality (MAPQ) below 20 were removed using Samtools v.1.3.1.^48^. Local realignment around indels and base quality score recalibration were applied with the Genome Analysis Toolkit (GATK 3.4.0)^49^. Duplicated reads were discarded using Picard MarkDuplicates v2.2.4 (https://broadinstitute.github.io/picard/). The MuTect2 algorithm was used to identify somatic mutations, including single nucleotide variants (SNVs), small insertions, and deletions (INDELs)^50^. False positive variant calls that originated from oxoG artifacts were excluded. All the variants were annotated using SnpEff & SnpSift v4.3,^51^ VEP^52^ and oncotator^53^. To identify a high-confidence list of putative somatic mutations, the following filtering steps were applied: (1) total allele count >=50 and variant allele frequency (VAF) > = 5%; (2) minor allele frequency (MAF) < 1% in gnomAD^54^ all and east Asian (EAS), and 1000 genome project^55^; (3) nonsynonymous SNVs or indels in coding regions; (4) exclude variants with “benign” or “likely-benign” of ClinVar clinical significance value. Copy number variations (CNVs) were detected using MuTect2 with default parameters. Fifty non-cancer samples (in-house data) were used as the panel of normal.

### Comparing with public data

Mutation frequencies of candidate genes were compared with those of the public database. We downloaded the clinical and mutation profile data of two major large-scale study, “Breast Invasive Carcinoma (TCGA, Nature 2012)”^56^ and “Breast Cancer (METABRIC, Nature 2012 & Nat Commun 2016)”^57^ from cBioPortal. (http://www.cbioportal.org). HER2-positive luminal B BC was selected by the “Positive” value of “ER Status” and “HER2 Status” in the clinical data from samples with both clinical and mutational data available. Fifty-two of 825 TCGA (6.3%) and 100 of 2368 METABRIC (4.2%) met the criteria. Extracted variants were annotated using VEP and further filtered by the same criteria used to identify the somatic mutations as descripted above.

### Validation of *MLH1* mutation by Sanger sequencing in tumor and matched normal samples

We performed Sanger sequencing of matched normal tissue to demonstrate whether *MLH1* mutations found in targeted sequencing were germline alterations as previously descripted^58^. Amplification was performed in a total volume of 20 μL containing 100ng-1μg of DNA from formalin-fixed and paraffin-embedded tissue using Maxime PCR PreMix (iNtRON Biotechnology, Korea). The primer sequence is shown in Supplementary Table 3. The PCR products were purified and analyzed using an ABI3730XL DNA analyzer (Appied Biosystem, Foster City, CA) for Sanger sequencing to validate the presence of each mutation.

### Molecular findings for microsatellite instability (MSI)

We performed an MSI analysis on paraffin-embedded tissues to evaluate their MSI status. The MSI status of the tumor samples was determined using the five-marker Bethesda panel (BAT25, BAT26, D5S346, D2S123 and D17S250)^59^. Polymerase chain reaction (PCR) products were run on an Qsep 100 DNA fragment analyzer (Bioptic Inc., Taiwan) and analyzed using Qsep 100 viewer (Bioptic Inc., Taiwan). Microsatellite instability was defined by the presence of different sized alleles in tumor DNA compared with the matched normal DNA sample. We classified the results into microsatellite instability-high (MSI-H), microsatellite instability–low (MSI-L) and microsatellite stable (MSS) in tumors according to Bethesda guidelines^60^.

### Immunohistochemical analysis

Immunohistochemical analysis for MLH1 protein was performed in 13 cases using the automated XT iVIEW DAB V.1 procedure on the BenchMark XT (Ventana, Tucson, AZ, USA), with incubation with MLH1 primary antibody (1:100, ES05, Dako) at 37 °C for 32 minutes. This was followed by a standard Ventana signal amplification and counterstaining with hematoxylin. Slides were mounted and examined by light microscopy. Only nuclear staining was considered for interpretation of data.

### Statistical analysis

The Pearson chi-squared test and the Fisher’s exact test were used for the analysis. The time to recurrence-free survival was defined from the day of first surgery until recurrence. All tests were two-sided, with *P* < 0.05 considered as statistically significant. All statistical analyses were performed using the SPSS software (SPSS Inc., Chicago IL, USA).

## Supplementary information

Supplementary figures

Supplementary tables

## Data Availability

Please contact the corresponding author for all data requests.
