# Supplementary material for: High prevalence of the MLH1 V384D germline mutation in patients with HER2-positive luminal B breast cancer
Source: Sci Rep. 2019 Jul 29;9:10966. doi: 10.1038/s41598-019-47439-3 (PMC6662670; doi:10.1038/s41598-019-47439-3)
Supplement: Supplementary file 1 — Supplementary figures [file 41598_2019_47439_MOESM1_ESM.pdf]

Supplementary Figures

High prevalence of the *MLH1 V384D* germline mutation in patients with HER2-positive luminal B breast cancer

Seung Eun Lee[1], Hye Seung Lee[1], Kyoung-Yeon Kim[2], Jung-Hoon Park[2], Hanseong Roh[2], Ha Young Park[3*] , Wan-Seop Kim[1*]

[1]Department of Pathology, Konkuk University Medical Center, Konkuk University School of Medicine, Seoul, Republic of Korea

[2] Precision Medicine Institute, Macrogen, Inc

[3]Department of Pathology, Busan Paik Hospital, Inje University College of Medicine, Busan, Korea

Corresponding authors: wskim@kuh.ac.kr, pmint00@naver.com

Supplementary Figure 1. Comparing mutation rate of *PIK3CA* and *TP53* with large-scale studies.

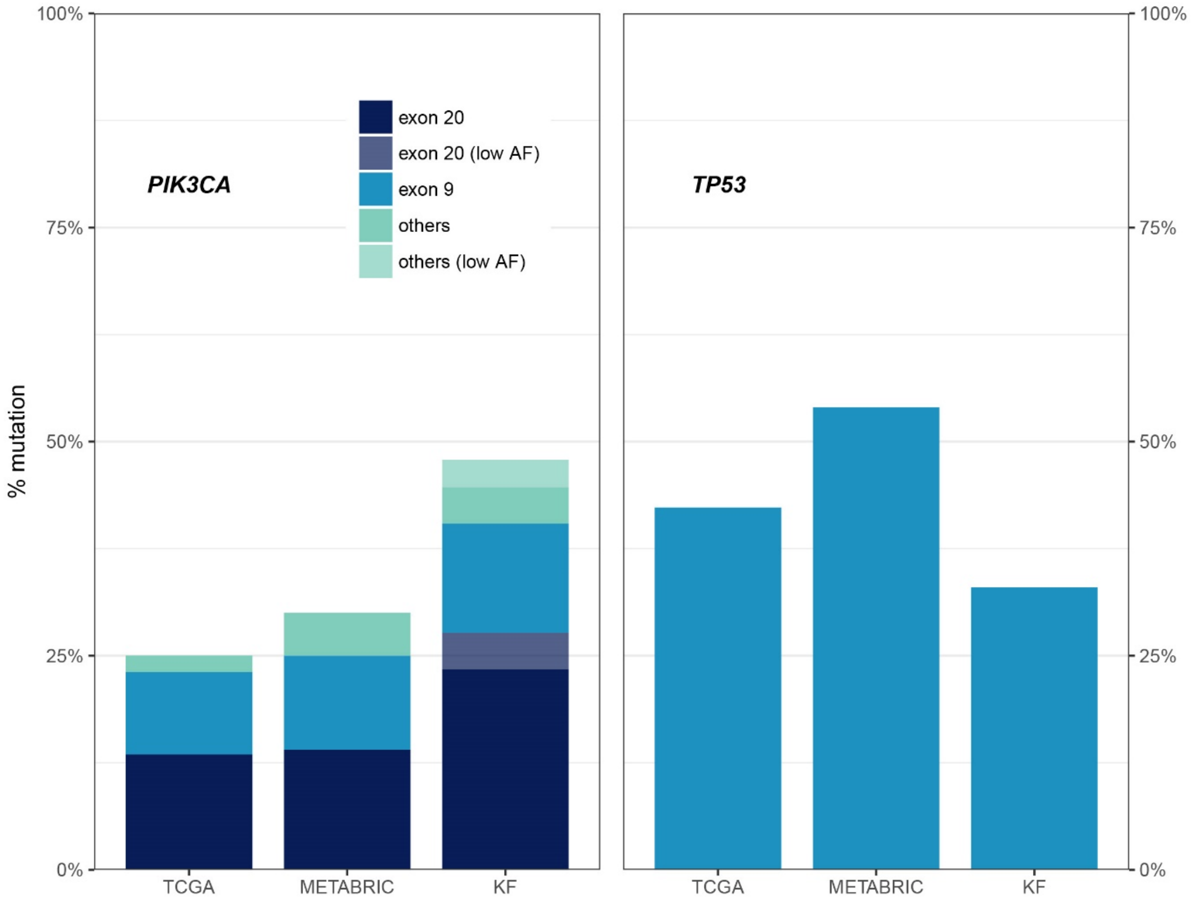

Bar plots represent mutation rate in HER-2-positive luminal B breast cancer of TCGA, METABRIC, and present study (KF). Different mutation loci of *PIK3CA* are distinguished by different colors.

Supplementary Figure 2. Details of *PIK3CA* mutations in three populations.

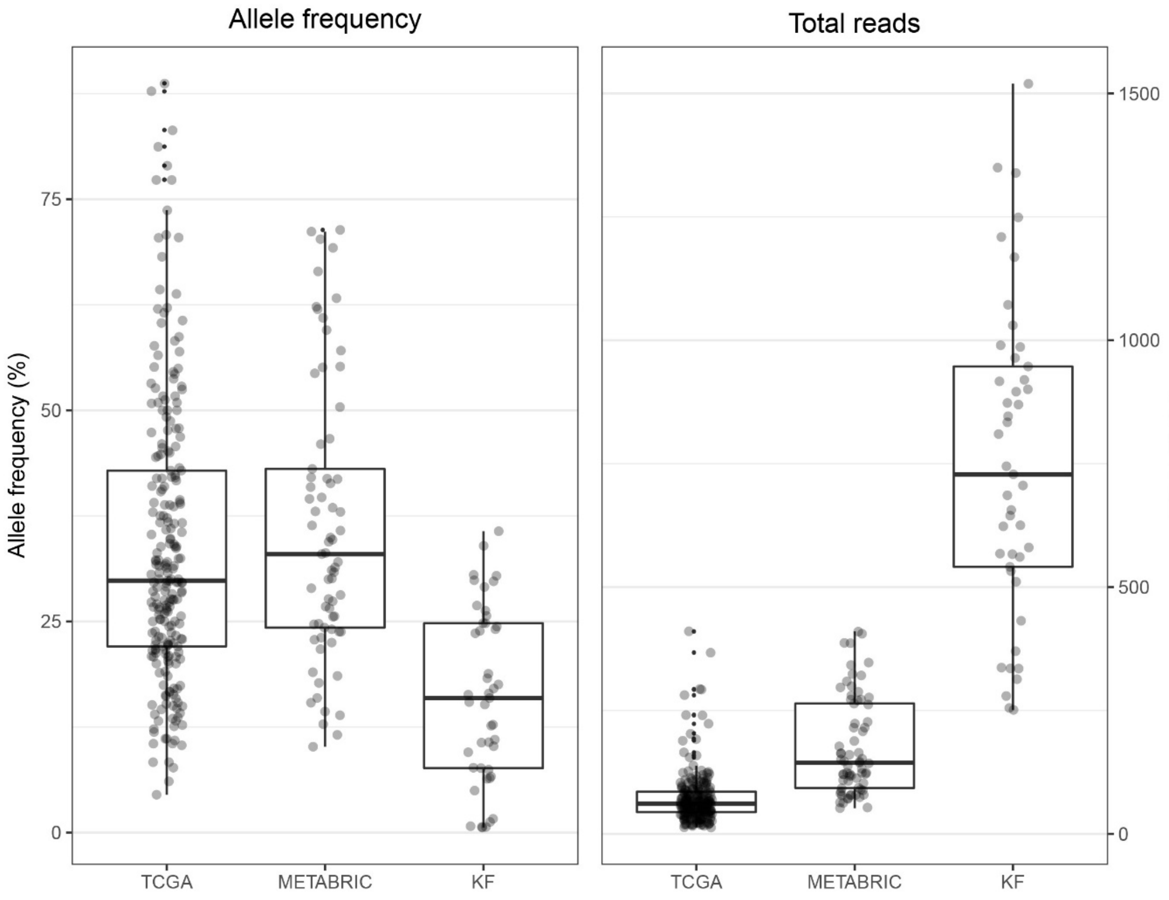

Variant allele frequencies and total reads of *PIK3CA* mutations of TCGA, METABRIC, and present study (KF) are summarized by bar plot, and each dot represent mutated cases.
